# Stabilized Reversed Polymeric Micelles as Nanovector for Hydrophilic Compounds

**DOI:** 10.3390/polym15040946

**Published:** 2023-02-14

**Authors:** Mariacristina Gagliardi, Agnese Vincenzi, Laura Baroncelli, Marco Cecchini

**Affiliations:** 1 NEST, Istituto Nanoscienze-CNR and Scuola Normale Superiore, Piazza San Silvestro, 56127 Pisa, Italy; 2 Institute of Neuroscience, National Research Council (CNR), Via Giuseppe Moruzzi 1, 56124 Pisa, Italy; 3 Department of Developmental Neuroscience, IRCCS Stella Maris Foundation, Viale del Tirreno 331, 56128 Calambrone, Italy

**Keywords:** reversed micelles, crosslinking, amphiphilic copolymers, hydrophilic cargo

## Abstract

Small hydrophilic drugs are widely used for systemic administration, but they suffer from poor absorption and fast clearance. Their nanoencapsulation can improve biodistribution, targeted delivery, and pharmaceutical efficacy. Hydrophilics are effectively encapsulated in compartmented particles, such as liposomes or extracellular vesicles, which are biocompatible but poorly customizable. Polymeric vectors can form compartmental structures, also being functionalizable. Here, we report a system composed of polymeric stabilized reversed micelles for hydrophilic drugs encapsulation. We optimized the preparation procedure, and calculated the critical micellar concentration. Then, we developed a strategy for stabilization that improves micelle stability upon dilution. We tested the drug loading and delivery capabilities with creatine as a drug molecule. Prepared stabilized reversed micelles had a size of around 130 nm and a negative z-potential around −16 mV, making them functional as a drug carrier. The creatine cargo increased micelle size and depended on the loading conditions. The higher amount of loaded creatine was around 60 μg/mg of particles. Delivery tests indicated full release within three days in micelles with the lower cargo, while higher loadings can provide a sustained release for longer times. Obtained results are interesting and encouraging to test the same system with different drug cargoes.

## 1. Introduction

Small molecules are the most common drugs in medical practice [1]. The roster of small drugs comprises a certain number of hydrophilic molecules. Based on the solubility limit in water, a drug can be defined as highly soluble (solubility > 1000 mg/mL), soluble (33–100 mg/mL), and sparingly soluble (10–33 mg/mL) [2]. Small hydrophilic molecules undergo rapid clearance upon administration, leading to a non-optimal biodistribution [3], and an impaired intracellular absorption due to their high polarity [4]. In the neuropsychiatric field, the blood–brain barrier also represents a physical obstacle for the delivery of hydrophilic active molecules to the central nervous system [5,6]. Briefly, although the systemic administration of small hydrophilics is relatively simple and safe, e.g., via intravenous injection, their pharmacodynamics and therapeutic efficacy could be at the very least non-optimal.

A promising strategy to improve the biodistribution and efficacy of such molecules is the encapsulation in a nanovector [7]. Nanovectors already tested for the delivery of hydrophilic small drugs are liposomes [1], extracellular vesicles [8], or core/shell nanoparticles [9]. All the mentioned nanocarriers have a compartmentalized structure to allocate the hydrophilic drug cargo. Polymeric micelles have a similar compartmentalized structure, and some great advantages, including easily functionalized and tuned in size and composition for best compliance as drug nanocarriers [10].

Micelles are self-assembled structures, composed of amphiphilic molecules, able to form emulsions between non-miscible solvents [11]. Commonly, ‘normal’ micelles are produced in water [12], while ‘reversed’ micelles are formed in non-polar liquids [13]. In both cases, the solvent represents the continuum phase while smaller amounts of non-miscible solvents are necessary to form the dispersed phase. The major difference between normal and reversed micelles is the arrangement of hydrophilic/hydrophobic functionalities throughout the structure [14]. The molecular arrangement involves the formation of a core/shell structure in which the shell has properties similar to those of the continuous phase and the core to those of the dispersed phase. Consequently, a normal micelle possesses a hydrophobic core and a hydrophilic shell, while the structure of an inverted micelle is arranged the other way around [15]. Owing to their capability to be customized to have a hydrophilic or hydrophobic core, micelles are interesting structures for the dispersion of molecules in solvents with opposite degrees of hydrophilicity/hydrophobicity. Amphiphilic multi-block polymers are suitable to obtain polymeric micelles and are also finely customizable to tune the ratio between the hydrophilic and the hydrophobic block, the overall molecular weight, and the end-chain functionalities [16].

Although they are considered interesting, normal polymeric micelles have relatively high critical micelle concentrations [17]. Thus, they need high amounts of polymer molecules to be formed and are poorly stable when diluted, e.g., after the administration in the host body. The critical micelle concentration, or the lower concentration of amphiphilic molecules to obtain micelles, is strictly related to the micelle stability to dilution to avoid their disaggregation [18]. Poorly stable micelles show a decreased blood circulation time and uncontrolled drug leakage after the administration. Moreover, normal micelles are more prone to encapsulate hydrophobic drugs [19]. Despite their limitations, many examples are reported in the literature describing the use of normal micelles as nanocarriers for the transport of drugs, nucleic acids, proteins, and contrast agents [20,21].

On the contrary, the reported applications of reversed micelles are less numerous and only concern the transport of proteins and peptides. To the best of our knowledge, polymeric reversed micelles are reported in only two works in the current literature. Koyamatsu et al. [22] reported the formulation of reversed micelles for the controlled release of erythropoietin protein in neutral or basic pH conditions. The micelles obtained needed to be coated to minimize the non-specific interactions with the plasmatic components. Jones and coworkers [23] demonstrated that subcutaneously administered reversed micelles, loaded with vasopressin, significantly increased the pharmacological effect of the active molecule in in vivo experiments. The studies mentioned focused on not fully stabilized reversed micelles that present the previously cited limitations, related to the disaggregation and the instantaneous leakage of the cargo. A suitable technique to stabilize the micellar structure is to provide crosslinking. Crosslinked structures possess intermolecular bridges that can interest the micelle core [24] or can be situated along the macromolecular backbone [25,26]. To date, there are no studies in the literature reporting the development of a system composed of crosslinked reversed micelles for the delivery of drugs.

With this work, we want to contribute to this field, reporting the preparation and the study of a system composed of stabilized reversed micelles for the encapsulation of hydrophilic molecules. The micellar structure was prepared from the amphiphilic di-block copolymer composed of a degradable hydrophobic block of poly(d,l-lactide-*co*-glycolide) and a water-soluble block of polyethylene glycol. The recipe to obtain reversed micelles with controlled size was optimized on the basis of a preliminary analysis. Then, we developed a chemical strategy to crosslink the reversed micelles and freeze the structure, to avoid micelle disaggregation upon dilution and other additional aggregation phenomena, typical of non-stabilized micelles. At the end, we assessed the capabilities of loading and release of the produced system using creatine, a small hydrophilic molecule of therapeutic relevance for a group of neurodevelopmental disorders [27,28], as a testbed.

## 2. Materials and Methods

All the materials, reagents, and solvents were purchased from Sigma Aldrich (St. Louis, MO, USA), if not otherwise stated.

### 2.1. Preliminary Study

#### 2.1.1. Selection of the Solvents

Solvents for the continuous and dispersed phases were selected to be compatible with all the procedures. The continuous phase should be non-polar or less polar than the dispersed phase, should be a good solvent for PLGA, and simple to be eliminated. The dispersed phase should be polar and compatible with the PEG block and hydrophilic drugs. Moreover, the dispersed phase should be non-miscible or poorly miscible with the continuous phase. Such requirements are met by dimethyl carbonate/water and chloroform/water pairs. Water is fully miscible in dimethyl carbonate (DCM) up to 3.32% in weight [27] and up to 0.08% in chloroform (CHL) [29,30].

We evaluated the formation of water vesicles in the selected solvents by mean of DLS analysis. Measurements were performed on the solvent/water mixtures with increasing amounts of water weight from 0 to 6% (Table 1). Samples were added to a quartz cuvette and measured at 20 °C. The viscosities at 20 °C used for this analysis are 0.664 and 0.563 mPa·s for DMC and CHL, respectively, while the refractive indexes are 1.3687 and 1.4459, respectively [31,32].

#### 2.1.2. Identification of the Optimal Polymer Concentration Range

The optimal concentration range for reversed micelles preparation was identified by measuring the size of micelles/vesicles formed in polymer solutions with decreasing concentrations after the addition of small amounts of water. We performed this analysis on polymer solutions with concentrations ranging from 10 to 2.5 mg/mL, in DMC and CHL. We fixed a molar water-to-polymer ratio, commonly indicated as *W*_0_ (Equation (1)), to 6, and followed the size of the particles formed through time. The analysis provided information not only on the effect of polymer concentration but also on the micelle stability with time after their formation.

(1) $$W_{0}=\frac{\left[water\right]}{\left[polymer\right]}$$

Prepared polymer solutions were added to a quartz cuvette, and then the fixed amount of water was added and the size of the particles were measured every 3 min over 90 min.

Results were combined with those obtained from the evaluation of water vesicles in the pure solvents. At that point, we decided to discard DMC as solvent for the reversed micelles preparation. Afterward, all the characterizations related to the preparation of reversed micelles were performed by using CHL as solvent.

### 2.2. Optimization of the Reversed Micelles Recipe

The optimization of the recipe was performed on non-stabilized reversed micelles. We calculated the critical micellar concentration, the best polymer concentration, and the best water-to-polymer molar ratio, with the aim to optimize micelle size, polydispersion, and concentration.

#### 2.2.1. Critical Micellar Concentration

The critical micellar concentration (CMC) is the concentration of surfactants above which micelles form and all additional surfactants added to the system become micelles [33]. In this work, CMC was calculated on micelle solutions obtained from polymer solution with decreasing concentrations, from 3.0 to 1.9 mg/mL. The concentration range was selected on the basis of the previous characterization. The starting micelle formulation was obtained from a polymer solution in CHL at the concentration of 3% *w/v* after the addition of a fixed amount of water (*W*_0_ = 6). Samples were measured by Dynamic Light Scattering (DLS, Malvern Panalytica Z-Zetasizer, Malvern UK) 30 min after the preparation, and then diluted with increasing volumes of CHL and measured after each dilution. DLS measures were used to evaluate the photon count, which is a parameter related to the colloid concentration. The photon count was then plotted against the polymer concentration and the CMC was calculated at the slope inversion point of the trends. Tests were performed in triplicate.

#### 2.2.2. Optimization of Size, Polydispersion, and Concentration

The final recipe was defined based on a further DLS experiment in which we systematically varied the polymer concentration and the *W*_0_. We analyzed three polymer concentrations in CHL (2.5, 3.3, and 5 mg/mL), and a *W*_0_ ranging from 5 to 34. To perform this test, we prepared the polymer solutions that were added to a capped quartz cuvette. Samples were previously measured every 2 min by DLS for 10 min to ensure that the starting polymer solutions were stable. Then, fixed amounts of water were progressively added to the cuvette, monitoring the micelle formation by DLS, acquiring data every 2 min for 10 min.

### 2.3. Synthesis of Stabilized Reversed Micelles

Stabilized reversed micelles were obtained from a modified mPEG-*b*-PLGA copolymer and by using the recipe optimized in the tests previously reported.

#### 2.3.1. Polymer Modifications

**Figure 1:**
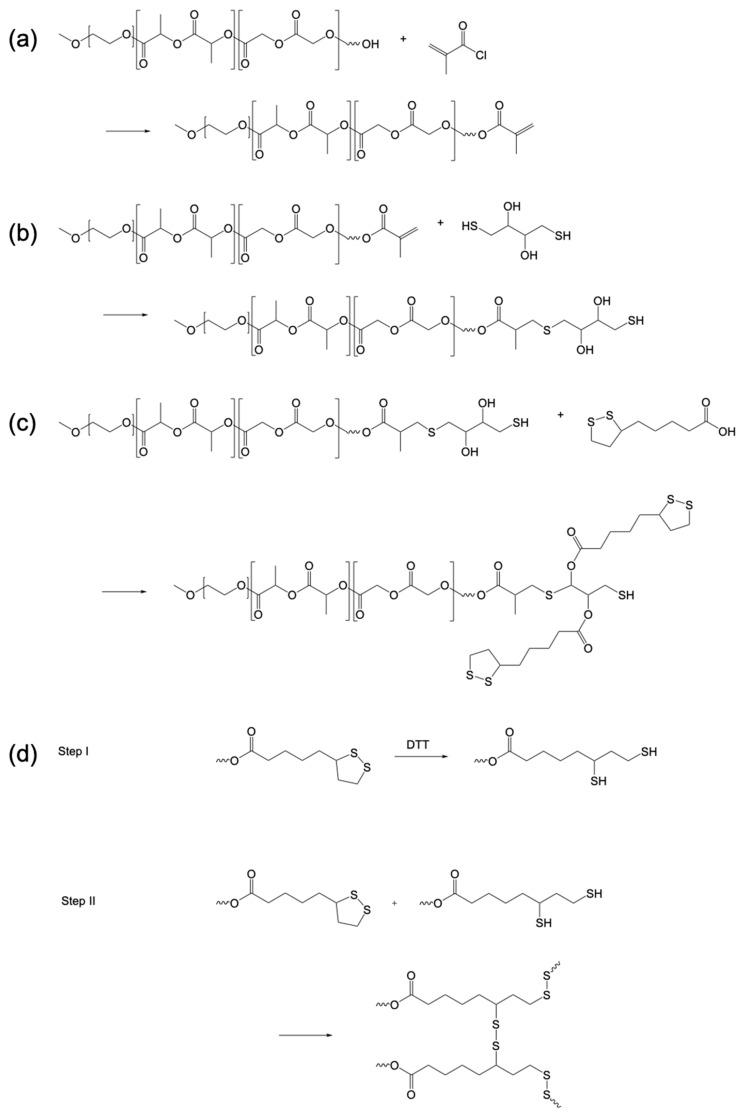
Chemical scheme of reversed micelles synthesis: (**a**) modification of the commercial mPEG-*b*-PLGA –OH terminated copolymer to obtain product **2**; (**b**) modification of product **2** by adding a terminal DTT unit to obtain the product **3**; (**c**) modification of the product **3** by adding two lipoic acid units per copolymer molecule to obtain the product **4**; (**d**) intermolecular crosslinking reaction to obtain the stabilized structure of the reversed micelles, Step I: reduction of a few LA units by DTT, Step II: reduction of LA units by –SH on the copolymer molecules.

The commercial mPEG-*b*-PLGA has a –OH termination (**1**) that can be modified to obtain the crosslinkable copolymer suitable for crosslinking (Figure 1). The chemical modification of the polymer was performed in three steps. In the first step, we added a terminal double bond to the PLGA end-chain through the reaction with methacryloyl chloride (MA), to obtain the ene-terminated polymer (**2**). Product **2** was further modified with dithiothreitol (DTT) to add two –OH functionalizable groups (**3**). Product **3** was modified with lipoic acid (LA) to add two crosslinkable sites (**4**).

##### Synthesis of Product 2

In a one-neck round-bottom glass reactor, 700 mg of **1** (51.9 nmol) was dissolved in 28 mL of CHL. Once the polymer was dissolved, 8.9 mg (72.6 nmol) of 4-dimethylaminopyridine (DMAP) was added and stirred for a few minutes. Then, 7.1 μL of MA (72.6 nmol) was added to the reactor. The reaction was stopped after 4 h, and then the product was dried under vacuum, washed three times with methanol, and finally dried under vacuum. Weight yield was 79% (553 mg).

^1^H-NMR (300 MHz, CDCl_3_): δ/ppm 5.2 (-OC**H**(CH_3_)COO-), 4.8 (-OC**H_2_**COO-), 4.2–4.5 (-C**H_2_**OC**H**O-), 1.6 (- OCH(C**H_3_**)COO-); for acryloyl end-functionalities, signals of geminal protons overlap with signals in the range 4.5–4.9 ppm, while the signal due to the COOC**H**=CH_2_ proton appeared at 6.7 ppm.

##### Synthesis of Product 3

Five hundred milligrams of **2** (37 nmol) was dissolved in 25 mL of acetone and slowly added to a mixture containing 20 mg of DTT (129.6 nmol) and 12.5 μL of ethylene diamine (EDA, 185.2 nmol) in 2.5 mL of acetone. The reaction was stopped after 24 h, and then the product was dried under vacuum, washed three times with methanol, and finally dried under vacuum. Weight yield was ~100% (~500 mg).

^1^H-NMR (300 MHz, CDCl_3_): δ/ppm 5.2 (-OC**H**(CH_3_)COO-), 4.8 (-OC**H_2_**COO-), 4.2–4.5 (-C**H_2_**OC**H**O-), 1.6 (- OCH(C**H_3_**)COO-); signals at 3.7 ppm (-C**H**OH-), 3.2 ppm (-CH_2_S**H**), 2.7 ppm (-C**H**H-), 1.6 ppm (-CH**H**-) attributed to DTT.

##### Synthesis of Product 4

In a one-neck round-bottom glass reactor, 500 mg of **3** (37 μmol) was dissolved in 62 mL of CHL. Once the polymer was dissolved, 71 mg of N-(3-dimethylaminopropyl)-N′-ethylcarbodiimide hydrochloride (EDCl, 370 μmol), 42.6 mg of N-hydroxysuccinimide (NHS, 370 μmol), 5 μL of triethylamine (TEA, 37 μmol), and 91.7 mg of LA (444.4 μmol) were added. The reaction was stopped after 24 h, and then the product was dried under vacuum, washed three times with methanol, and finally dried under vacuum. Weight yield was 66% (329 mg).

^1^H-NMR (300 MHz, CDCl_3_): δ/ppm 5.2 (-OC**H**(CH_3_)COO-), 4.8 (-OC**H_2_**COO-), 4.2–4.5 (-C**H_2_**OC**H**O-), 3.7 (-C**H**OH-), 3.2 (-CH_2_S**H**), 2.7 (-C**H**H-), 1.6 (-CH**H**-) and (- OCH(C**H_3_**)COO-); signal at 3.3 ppm is attributed to lipoic ring.

#### 2.3.2. Reversed Micelles Synthesis

The synthesis of reversed micelles consists in the crosslinking of **4** after their self-assembly. To do this, we added after the self-assembly a catalytic amount of DTT as reducing agent to start the reaction. The crosslinking reaction proceeds as a cascade: in the first step (Figure 1d, Step I), the DTT molecule reduces only a few amounts of LA units in the copolymer; in the following steps (Figure 1d, Step II), the free –SH units can reduce the LA units and react to form a disulfur —S—S— intermolecular bridges.

Twenty milligrams of **4** (1.5 nmol) was dissolved in 7 mL of CHL in a closed-cap glass vial; 550 μL of water (30.5 μmol) was added to the polymer solution, and mildly stirred for 1 min. After 1.5 h, 0.8 mg of DTT (5.2 nmol) was added. The mixture is maintained in the closed-cap vial for 48 h without any stirring. At the end of the procedure, the product was dried under vacuum to eliminate the organic solvent, and then washed with ethanol three times to eliminate the residual reagents and solvent. After purification, micelles were resuspended in a trehalose solution (100 mg/mL), and then transferred to eliminate the residual non-crosslinked copolymer. The resuspended formulation was stored at −20 °C until the use. Synthesis was performed in triplicate. Stabilized reversed micelles were measured by DLS to evaluate the diameter, the z-potential, and the stability to dilution. The stability to dilution test was performed by measuring the size and the mean photon count of micelle suspensions with decreasing concentrations. The first measure was performed on a suspension with a concentration of 12.5 mg/mL (gravimetrically evaluated); the solution was then progressively diluted until the mean photon count was above 100 kcps, to ensure a reliable measure.

### 2.4. Drug Loading and Delivery

The capability of stabilized reversed micelles to entrap a hydrophilic cargo was tested using creatine (Mw 131.1 Da, solubility in water 14 mg/mL at 20 °C [34]). The creatine monohydrate molecule was loaded within micelles by absorption. Fifteen milligrams of crosslinked reversed micelles, suspended in a trehalose solution (trehalose concentration 100 mg/mL, reversed micelles concentration 0.1 mg/μL) was diluted with 300 μL of DMC. After dilution, 100 μL of creatine solution in PBS at different concentration was added. The selected concentrations of creatine solutions were: 1, 2, 3, 4, and 5 mg/mL. The mixture was stored at 4 °C for 48 h, and then the two phases were manually separated, the non-dissolved water phase was stored to measure the residual creatine concentration, while the organic solvent was evaporated under vacuum. Dried micelles were washed with 1 mL of PBS to eliminate the creatine remaining on the surface, and then resuspended in a trehalose solution (45 μL) and stored at −20 °C for drug delivery tests.

Creatine encapsulation was evaluated by UV-Vis spectroscopy. Fifteen microliters of the supernatants withdrawn at the end of the loading was diluted in 600 μL of PBS and added to a quartz cuvette for absorbance readings. The UV spectrum was acquired in the range from 200 to 400 nm, and the signal at 205 nm [35] was used to calculate creatine concentration. Absorbances were elaborated upon a calibration curve obtained from creatine solutions in PBS, in a concentration range from 8 to 82 μg/mL.

Creatine delivery tests were performed on loaded stabilized reversed micelles. Five microliters of micelle resuspension in trehalose, corresponding to 0.2–0.3 mg of RMs for each tested sample, was added to plastic vials, and then diluted with 100 μL of PBS (pH 7.4) and maintained at 37 °C for drug delivery tests. Delivery test conditions were selected to be similar to the physiological environment, and also to maintain the creatine molecule’s stability at neutral pH [34]. After fixed times, from 1 to 120 h, samples were centrifuged and the supernatant was used for the creatine quantification via UV-Vis spectroscopy. Samples were measured without any previous dilution and analyzed by using the calibration curve previously described. The amount of released creatine was normalized on the actual amounts of micelles used for the delivery test. The actual micelle concentrations in samples obtained after creatine loading were calculated via DLS measures. This quantification is possible because the mean photon count rate is proportional to the nanoparticle number concentration [36]. To do this, micelle samples with different concentrations, from 6.3 to 12.5 mg/mL (evaluated gravimetrically), were measured by DLS, thus acquiring the mean photon count (kcps). Then, the calibration curve was obtained and values of the mean photon count vs. micelle concentration (mg/mL) were reported.

Creatine loading tests and delivery tests were performed in triplicate.

### 2.5. Statistical Analysis

Data are reported as the average value ± SD for technical replicates, while average value ± SE for data are aggregated from replicated experiments. Error bars reported in the figures were calculated with the same method, if not otherwise stated.

## 3. Results

### 3.1. Preliminary Setup

**Figure 2:**
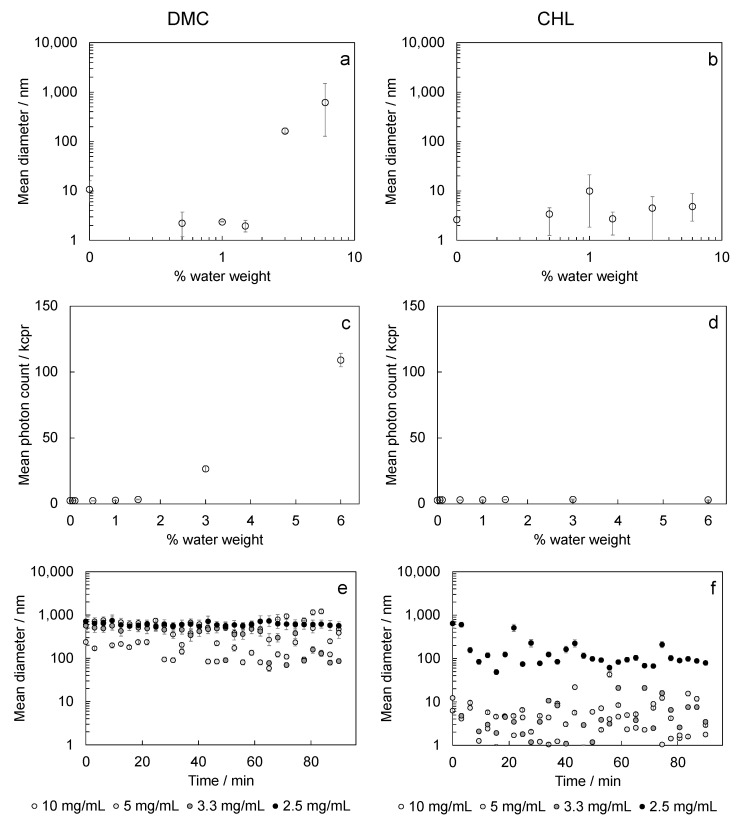
Preliminary analysis performed to select the best solvent for preparation of reversed micelles: evaluation of water vesicles in the selected solvent by varying the percent water weight ((**a**) DMC, (**b**) CHL); mean photon counts obtained in water vesicles measurements ((**c**) DMC, (**d**) CHL); preliminary evaluation of suitable polymer concentrations by measuring the size of vesicles/micelles formed during time ((**e**) DMC, (**f**) CHL).

Water-in-solvent vesicles (Figure 2a,b), which are involved in the determination of the micelle core, were analyzed in a percent weight range of water that we considered suitable for our procedure. Results obtained for DMC show that, out of the water solubility range (>3% *w*/*w*), some very large vesicles were formed. On the contrary, measures on CHL/water samples did not show the presence of vesicles. The measured values in samples in which vesicles were not formed were related to a background signal attributed to the solvent, and the mean photon count was less than 5 kcps; the same count increases when vesicles were formed (Figure 2c,d).

Measures performed to identify a polymer concentration range in dimethyl carbonate-based samples at the fixed W_0_ indicated that the formed micelles were too large for polymer concentrations of 2.5 and 10 mg/mL, which were quite unstable for intermediate concentrations (Figure 2e). CHL-based samples at fixed *W*_0_ gave quite stable and measurable micelles only for the lowest polymer concentration (Figure 2f).

Based on the results obtained, we decided to discard DMC as a solvent for micelle preparation and to further investigate the micelle formation by using polymer solutions in CHL, and to keep the polymer concentration lower than 5 mg/mL.

### 3.2. Optimization of the Reversed Micelles Recipe

**Figure 3:**
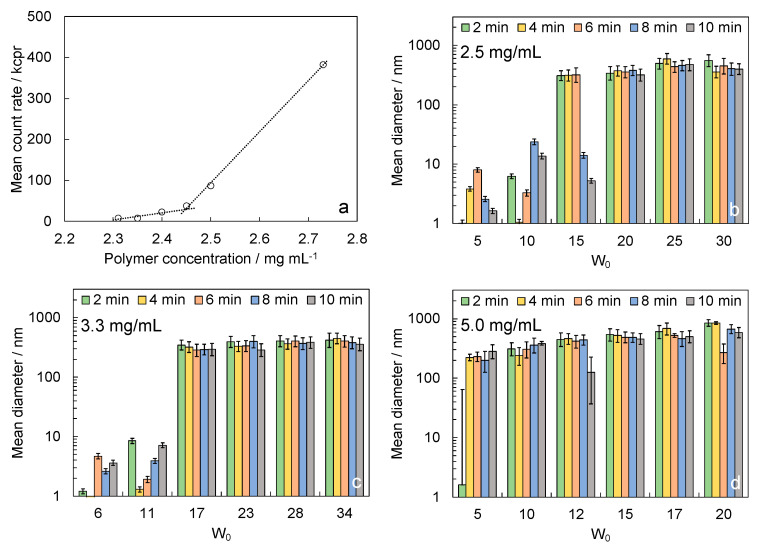
Optimization of the reversed micelles recipe: (**a**) calculation of the critical micellar concentration from DLS analysis—the plot reports one of the three performed experiments as an example; reversed micelle size, measured by DLS, at 2, 4, 6, 8, and 10 min after the addition of water to the polymer solution with concentrations (**b**) 2.5, (**c**) 3.3, and (**d**) 5.0 mg/mL.

The CMC, calculated starting from polymer solution in CHL in three independent experiments, resulted in 2.5 mg/mL ± 0.1 mg/mL (Figure 3a). This was intended to be the lower concentration needed to obtain micelles with the fixed *W_0_.* Taking the previous experiments in consideration, the following experiments were focused on samples obtained from polymer solutions in the concentration range from 2.5 to 5.0 mg/mL.

DLS measurements through time of reversed micelles formed from different concentration polymer solutions by varying *W*_0_ (Figure 3b–d) indicated that polymer concentration strongly affects the final diameter of the reversed micelles. In particular, the higher concentration tested (5 mg/mL) gave particles that were too large, and then it was discarded. Tests performed with solutions with polymer concentrations of 2.5 and 3.3 mg/mL were both suitable for the proposed applications, giving reversed micelles with a diameter lower than 300–400 nm. From this analysis, W_0_ around 20 was selected.

On the basis of such results, we selected the intermediate concentration of 2.9 mg/mL to perform the synthesis of stabilized reversed micelles.

### 3.3. Stabilized Reversed Micelles Characterization

**Figure 4:**
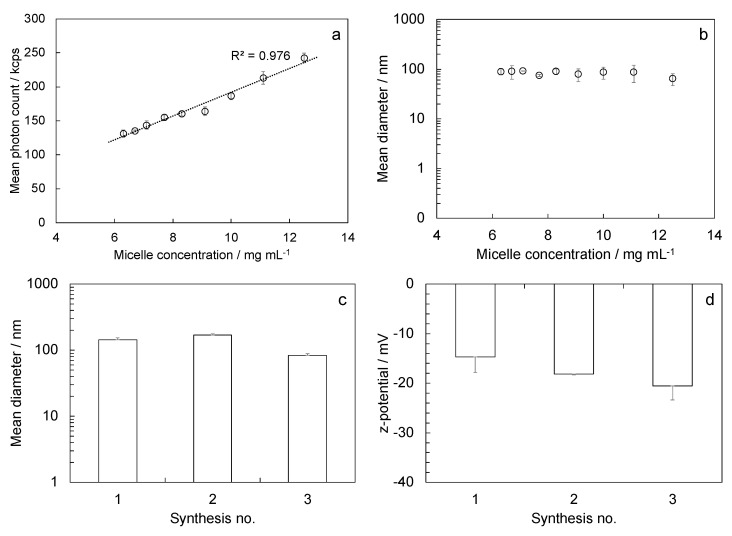
Characterization of stabilized reversed micelles: (**a**) mean photon count and (**b**) mean diameter calculated in dilution tests; (**c**) mean diameter and (**d**) z-potential evaluated in three repeated syntheses.

We performed a dilution test to evaluate the stability to dilution of stabilized reversed micelles and confirm the crosslinking that occurred. Starting from the higher micelle concentration and diluting, the mean photon count decreased linearly (Figure 4a), while the measured diameter of micelles did not vary (Figure 4b).

Synthesized micelles showed a mean diameter of 132 nm ± 26 nm, calculated from nine measures from three different syntheses. Size variability through replicated syntheses was low, indicating that the procedure is reliable. The mean PDI was 0.23 ± 0.12; the mean z-potential result was −15.6 mV ± 1.7 mV.

### 3.4. Drug Loading and Delivery

**Figure 5:**
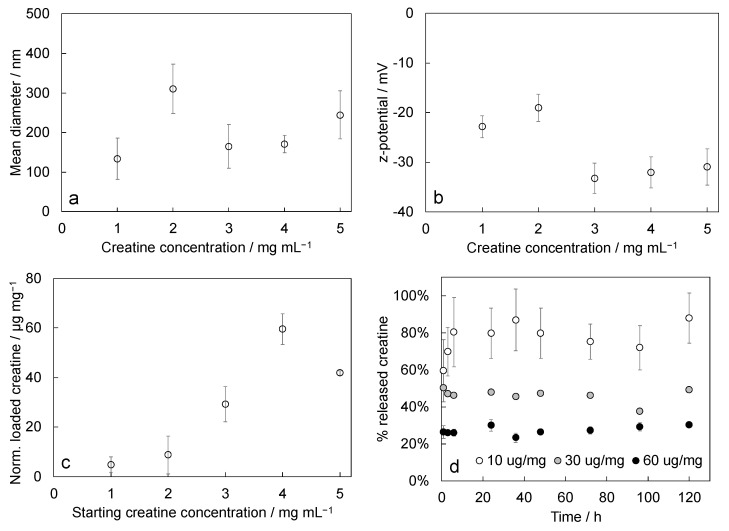
Characterization of creatine-loaded stabilized reversed micelles: (**a**) mean diameter and (**b**) z-potential of micelles loaded with different starting amounts of creatine; (**c**) amount of loaded creatine (μg) by varying the starting creatine solution concentrations, normalized to the actual amounts of micelles (mg); (**d**) percent of released creatine through time (μg) with respect to the actual amounts loaded, in the legend formulations are labeled according to the amount of creatine loaded per mg of RMs. In plots (**a**–**c**), the *x*-axis reports the starting concentration of the creatine solution used during the loading procedure.

Stabilized reversed micelles size after creatine loading showed an increase in the mean diameter of around 56% with respect to the mean value measured on empty micelles (Figure 5a). The mean diameter of creatine-loaded micelles was 205 nm ± 25 nm. Size increase followed a trend with the amount of loaded creatine, increasing linearly from 1% to 190% by increasing the creatine cargo. The absolute value of the z-potential increased around 55% after loading, resulting in a mean value of −27.6 mV ± 1.2 mV (Figure 5b). There was no particular trend relating the z-potential change with the amount of loaded creatine. The cargo molecule has a null formal charge [37], thus a trend was not expected.

The amount of loaded creatine by varying the starting concentration of the solution used for the loading procedure (Figure 5c) increased up to the experiment performed with the solution at 4 mg/mL. The systems with the two lower creatine loadings produced similar results; thus, we decided to discard the lower one. Samples prepared for creatine delivery tests contained amounts of creatine ranging from 2 to 18 μg, on the basis of the calculated RM amount and creatine loading per mg of RMs. With these amounts of creatine, the theoretical maximum concentration of creatine in the delivery medium was in the range from 20 to 180 ug/mL. Creatine delivery kinetics (Figure 5d) indicated that differences in released amounts occurred only between samples loaded with the lower creatine cargo, while no differences were detected for samples obtained by loading with creatine solutions with concentrations 3 and 4 mg/mL. Micelles loaded with the lowest amount of creatine released 88% ± 14% of the cargo, while samples loaded with the creatine solutions with concentrations 3 and 4 mg/mL released 49% ± 16% and 30% ± 1%, respectively. To verify that the slow release at late times was not affected by the saturation of the delivery medium, we checked if the perfect sink condition was maintained throughout the test time. The perfect sink condition occurred when the amount of solute delivered was lower than 10–20% or even 30% of the maximum solubility of the solute in the dissolution medium [38]. The solubility limit of creatine in water is 14 mg/mL; 10% of the solubility limit corresponds to 1.4 mg/mL. In these tests, the maximum theoretical concentration of creatine was 180 ug/mL. This value is significantly lower than 1.4 mg/mL; thus, the perfect sink condition is ensured. We can therefore conclude that the slow delivery kinetics after three days was not due to the saturation of the delivery medium.

## 4. Discussion

To form a nanovector for the delivery of active molecules that avoids potentially harmful bioaccumulation, we selected a biocompatible and degradable polymer that is well tolerated by the host body. We opted for the copolymer poly(d,l-lactide-*co*-glycolide) (PLGA) that is a well-known material, already approved by the FDA for drug delivery applications [39]. This copolymer is fully hydrophobic and therefore not suitable for micelle preparation. By adding a hydrophilic block covalently linked to the PLGA, the resulting di-block copolymer is amphiphilic and can thus form micelles. We selected as hydrophilic block the polymer polyethylene glycol (PEG), which is also already approved by the FDA [40]. We defined the overall molecular weight of the polymer and the weight ratio between the hydrophobic and the hydrophilic block on the basis of the Griffin’s theory for non-ionic surfactants [41]. According to the cited theory, we calculated the hydrophilic–lipophilic balance (HLB) of some commercially available PEG-PLGA di-block copolymers. The parameter HLB, given by the ratio between the molecular weight of the hydrophilic block and the hydrophobic block multiplied by 20, is the quantification of the degree to which the macromolecule is hydrophilic or hydrophobic. The HLB parameter arbitrary scales from 0 (completely hydrophobic) to 20 (completely hydrophilic). Polymers used in the preparation of reversed micelles are water-in-oil emulsifiers. According to Griffin’s scale, such molecules have HLB in the range 3–6. We selected our copolymer among the commercially available methoxy-polyethylene glycol-*block*-poly(d,l-lactide-*co*-glycolide) (mPEG-*b*-PLGA) from Sigma Aldrich. The selected copolymer has a 2 kDa PEG block and a hydroxy-terminated 11.5 kDa PLGA block, with a lactide-to-glycolide molar ratio of 50/50. The HLB for this polymer is 3.0, resulting in a highly hydrophobic material.

The extensive preliminary analysis performed during the setup of the system showed that the preparation of polymeric reversed micelles was affected by several parameters. Parameters considered in this work were the pair of solvents selected, their relative volumes, the concentration of the selected polymer and its HLB, and the molar ratio between dispersed solvent and polymer. We can assert that none of these parameters can be neglected when aiming to control self-assembly of reversed micelles. The optimized recipe, defined on the basis of the preliminary analysis, allows for strictly controlling the size of the obtained reversed micelles.

The proposed chemical modification of the polymer is suitable for the application and a successful crosslinking is obtained as the dilution tests demonstrated. The final size of stabilized reversed micelles is compatible with potential applications as drug delivery carriers, for intravenous [42] or nasal [43] administration. The loading of active molecules in the reversed micelle core causes an increase in micelle size. The increase in micelle diameter is related to an effective cargo loading because the volume of the micelle core increases for the presence of a cargo. Despite the increase in size, the micelles remain suitable for the proposed applications. All the measured stabilized reversed micelles, either empty or creatine-loaded, show a negative z-potential and a limited PDI, meeting the criteria for administration [44]. The increase in encapsulation capacity with increasing concentrations of the creatine solution used during micelle loading is a desirable behavior for the proposed system. The proposed characterization provided a precise indication of the optimal concentration range to obtain an effective creatine loading, between 2 and 4 mg/mL. The lower concentration tested (1 mg/mL) gave an entrapment that was too low, while the higher concentration (5 mg/mL) was less effective.

This innovative system for creatine delivery might be a game changer for the cure of creatine deficiency syndromes, a family of three inherited metabolic disorders caused by mutations in the genes encoding for the synthetic enzymes of creatine (L-arginine:glycine amidinotransferase, AGAT, and S-adenosyl-L-methionine:N-guanidinoacetate methyltransferase, GAMT) or the transporter responsible for its cellular uptake (CRT). These neurodevelopmental conditions share a common clinical picture presenting with low creatine levels, developmental delay, intellectual disability, autistic-like behavior, epilepsy, but also a plethora of non-neurological symptoms, including heart, muscle, and gastrointestinal problems [45,46,47,48]. Since dietary supplementation of creatine leads to the attenuation of symptoms in AGAT (AGAT-D) and GAMT deficiency (GAMTD-D, [47,48]), and fails to restore creatine levels in CRT deficiency (CRT-D) [27], our polymeric nanovectors might represent an important tool to i) optimize the biodistribution and half-life of creatine in AGAT-D and GAMTD-D patients; ii) devise an effective therapeutic strategy for CRT-D. Indeed, loading creatine in micelles would allow the cellular uptake of the molecule even in absence of its specific transporter.

Importantly, the results of this study are not limited to creatine, but can also give indications for the loading of other molecules. The loaded creatine is rapidly released from micelles with the smaller cargo, reaching a complete release after three days. Micelles containing larger creatine cargoes have a moderate release that does not end in the time span analyzed in this work. Trends indicate a slow but increasing release of creatine; thus, we can foresee a larger amount of creatine released at late times. Moreover, the carrier is composed of a degradable PLGA block, and its degradation will lead to the complete release of the cargo at long times. The creatine delivery over five days indicates that a sustained release could be obtained only for the higher loadings. These data indicate that the system could have some further exploitations after few adaptations.

The production procedure is highly reliable but an additional parameter that can be optimized could by the time between the beginning of the self-assembly process and the addition of the reducing agent activating the crosslinking. This optimization can promote further control on micelle polydispersity.

The proposed system should be able to encapsulate different kinds of hydrophilics, regardless the size of the cargo molecule. Thus, another interesting aspect is the analysis of loading and delivery capabilities by varying the molecular weight of the cargo.

Interestingly, the surface chemistry of stabilized reversed micelles can be further decorated to add specific targeting properties. The conjugation with a ligand (e.g., a targeting peptide) can be a successful strategy to implement a targeting property to the system. For instance, a potential ligand targeting the nanovector to the brain is the peptide Angiopep-2, which is already known for its capability to penetrate the blood–brain barrier [49]. Considering the high complexity of the brain tissue and the relative impermeability of the blood–brain barrier, this technological advancement might represent a fundamental step in the therapeutic development of various brain disorders.

## 5. Conclusions

We proposed a consistent method for the preparation of stabilized polymeric reversed micelle to be used for the encapsulation of hydrophilic molecules. We tested the system with creatine, a small hydrophilic cargo, and evaluated the release kinetics starting from different amounts of loaded cargoes. Results are very interesting and push toward further exploitations of the proposed vector. In a future work, we will demonstrate that the proposed nanovector is also suitable for the delivery of large hydrophilic molecules toward the brain.

## 6. Patents

The work reported in this manuscript is patent pending (Reversed micelles for delivery of hydrophilic drugs, ref. 102022000014791, submission date July 2022).

## Figures and Tables

**Table 1:** Water weight percent of samples measured to evaluate water-in-solvent vesicle size and amounts of water (μg mL^−1^) added to the selected solvent.

Water Weight/%	Water Amount Added to DMC/μg mL^−1^	Water Amount Added to CHL/μg mL^−1^
0.05	0.7	0.5
0.10	1.5	1.1
0.50	7.5	5.4
1.00	15.1	10.8
1.50	22.7	16.3
3.00	46.1	33.1
6.00	95.1	68.3

## Data Availability

Datasets generated during the study will be freely available after publication.
